# Reactive Oxygen Species Damage Bovine Endometrial Epithelial Cells via the Cytochrome C-mPTP Pathway

**DOI:** 10.3390/antiox12122123

**Published:** 2023-12-16

**Authors:** Pengjie Song, Mingkun Sun, Chen Liu, Jianguo Liu, Pengfei Lin, Huatao Chen, Dong Zhou, Keqiong Tang, Aihua Wang, Yaping Jin

**Affiliations:** Key Laboratory of Animal Biotechnology of the Ministry of Agriculture, College of Veterinary Medicine, Northwest A&F University, Xianyang 712100, China; songpengjie@nwafu.edu.cn (P.S.);

**Keywords:** endometritis, mitochondria damage, ROS, cyclophilin D (CypD), mitochondrial permeability transition pore (mPTP), apoptosis

## Abstract

After parturition, bovine endometrial epithelial cells (BEECs) undergo serious inflammation and imbalance between oxidation and antioxidation, which is widely acknowledged as a primary contributor to the development of endometritis in dairy cows. Nevertheless, the mechanism of oxidative stress-mediated inflammation and damage in bovine endometrial epithelial cells remains inadequately defined, particularly the molecular pathways associated with mitochondria-dependent apoptosis. Hence, the present study was designed to explore the mechanism responsible for mitochondrial dysfunction-induced BEEC damage. In vivo, the expressions of proapoptotic protein caspase 3 and cytochrome C were increased significantly in dairy uteri with endometritis. Similarly, the levels of proapoptotic protein caspase 3, BAX, and cytochrome C were markedly increased in H_2_O_2_-treated BEECs. Our findings revealed pronounced BEEC damage in dairy cows with endometritis, accompanied by heightened expression of cyto-C and caspase-3 both in vivo and in vitro. The reduction in apoptosis-related protein of BEECs due to oxidant injury was notably mitigated following N-acetyl-L-cysteine (NAC) treatment. Furthermore, mitochondrial vacuolation was significantly alleviated, and mitochondrial membrane potential returned to normal levels after the removal of ROS. Excessive ROS may be the main cause of mitochondrial dysfunction. Mitochondrial permeability transition pore (mPTP) blockade by cyclophilin D (CypD) knockdown with CSA significantly blocked the flow of cytochrome C (cyto-C) and Ca^2+^ to the cytoplasm from the mitochondria. Our results indicate that elevated ROS and persistent opening of the mPTP are the main causes of oxidative damage in BEECs. Collectively our results reveal a new mechanism involving ROS-mPTP signaling in oxidative damage to BEECs, which may be a potential avenue for the clinical treatment of bovine endometritis.

## 1. Introduction

Endometritis is an inflammation of the endometrial lining, which occurs in dairy cows after calving [1]. Up to 40% dairy cows suffer from postpartum uterine disease [2]. Uterine diseases lead to infertility by impairing the function of the endometrium [3]. The pathogenesis of endometritis in cows is multifactorial, involving various factors such as bacterial infections, poor uterine involution, and oxidative stress [4,5]. Oxidative stress, an imbalance between the production and detoxification of ROS, has been implicated in the pathogenesis of endometritis [6]. In our previous study we found that an imbalance between oxidation and antioxidant in uterine endometritis [7]. Oxidative stress is one of the main causes of inflammation in BEECs [5]. Bovine uteri are composed of three layers, which are divided into the endometrial layer, muscular layer, and serosal layer [8]. The endometrium specifically serves as the site for embryo implantation. In cases of endometritis, the endometrium becomes congested and edematous, leading to the accumulation of inflammatory exudate [9]. That is not favorable for the upward movement of sperm, making it challenging for the embryo to implant successfully in the inflamed endometrium and increasing the risk of miscarriage [10]. Apoptosis is programmed cell death, crucial for the maintenance of tissue and organ homeostasis [11]. Following receipt of apoptotic signals, progressive alterations ensue within the cellular framework, encompassing impaired mitochondrial function, cytochrome c release, and activation of caspase-9 and caspase-3 [12]. Cytochrome c leakage is perceived as one of the earliest events exhibited during the execution of cell apoptosis. Subsequently, cytosolic cytochrome c engages apoptotic protease activating factor 1 (Apaf-1) to form a complex, thereby facilitating caspase-9 activation [13]. Once activated, caspase-9 orchestrates the activation of caspase-3, culminating in the ultimate induction of cell apoptosis [13]. ROS play a role in regulating the NLRP1 inflammasome [14,15], and are associated with a variety of disorders, including cardiovascular, neurodegenerative, and inflammatory diseases [16,17,18]. Effective management of oxidative stress involves maintaining a delicate balance between ROS production and antioxidant defenses, which comprise enzymes such as SOD1, CAT, and GPX1 [19]. Diminished expression of antioxidases indicates a reduced antioxidant capacity whereas excessive generation of ROS overwhelms the system’s ability to neutralize and eliminate them, resulting in oxidative stress that contributes to the development of diseases [20]. ROS act directly to damage proteins and nucleic acids, and disrupt mitochondrial Ca^2+^ homeostasis [21]. A recent study indicates icariin protects endometritis from oxidative damage by inhibiting TLR4-associated NF-κB pathways [22]. Dexamethasone directly reduced the generation of ROS by uterine PMN that would protect the endometrium from tissue damage by excessive extracellular ROS [23]. Elevated levels of ROS also impair key mitochondrial enzymes such as NADH dehydrogenase, cytochrome c oxidase, and ATP synthase, ultimately halting mitochondrial energy production. Notably, research in mammals has consistently shown a significant connection between oxidative stress and endothelial dysfunction [24,25]. Furthermore, ROS may facilitate mitochondrial permeability transition by oxidizing thiol groups on the adenine nucleotide translocator, which is believed to be part of the mPTP [26].

CypD, an enzyme functioning as a peptidyl-prolyl isomerase, resides in the mitochondrial matrix and plays a critical role in the formation and regulation of the mPTP. [27]. In addition, CypD has been identified as a crucial factor in mediating cell death mechanisms associated with various inflammation diseases [28,29]. Typically, diseases induced by oxidative stress are characterized by a decrease in ATP levels and an increase in calcium levels [30]. The mPTP is an indiscriminate and calcium-dependent channel complex situated within mitochondria that functions primarily to maintain the balance of the mitochondrial respiratory chain [31]. Increased calcium levels trigger persistent mPTP opening mediated by CypD, leading to cell death [27,32] and the subsequent release of ROS produced by mitochondria and cytochrome-C into the cytoplasm [33]. Research has suggested that mPTP opening is observed in erastin-treated cancer cells, as evidenced by VDAC-1 and Cyp-D association, mitochondrial depolarization, and cytochrome C release [33].

Mitochondria, as the powerhouse of cellular activity, play a crucial role in energy metabolism and must be in proper functional state to generate the necessary energy for basic cellular functions, including proliferation [34]. Structurally, mitochondria consist of four compartments: the outer membrane, the inner membrane, the intermembrane space, and the matrix [35]. There are numerous proteins present on and between these membrane structures that contribute to the synthesis of adenosine triphosphate (ATP). The mitochondrial respiratory chain complexes encompass five complexes that catalyze the phosphorylation of adenosine diphosphate to ATP [36]. Mitochondrial damage leads to a decrease in ATP synthesis. Under conditions of oxidative stress, ROS directly damage Ca^2+^-regulating proteins, disrupting Ca^2+^ homeostasis. Mitochondria play a crucial role in cellular respiration, producing ATP that is essential for maintaining normal cellular functions. When mitochondria become dysfunctional, they may generate excessive ROS which causes oxidative stress and damage to cellular structures.

In this study, we investigated the effects of mitochondrial dysfunction on BEEC damage to help clarify the mechanism underlying the development of bovine endometritis and provide a basis for strategies to improve embryo implantation in dairy cattle.

## 2. Materials and Methods

### 2.1. Animals and Ethics Statement

All experiments related to dairy cows were performed according with the guidelines of Animal Research Institute Committee at Northwest A&F University. Our study exclusively utilized uterine samples obtained from 3 to 5-year-old Holstein–Friesian cows that had undergone 2–4 parities and were within 40–60 days postpartum. These uteri were categorized into healthy and endometritis groups based on cytological and histopathological diagnoses as outlined previously [7]. The collected uterine tissue samples were then divided into two portions: one part was preserved in 4% paraformaldehyde for subsequent histopathological analysis, while the other part was promptly frozen in liquid nitrogen for subsequent total protein extraction.

### 2.2. Cell Culture and Treatment

BEECs were isolated from healthy uteri as previously described [28]. BEECs were cultured in Dulbecco’s modified Eagle’s medium (DMEM)/F12 (Hyclone, Logan, UT, USA) supplemented with 10% fetal bovine serum (FBS; ZETA, Lower Gwynedd Township, PA, USA). The culture was maintained at 37 °C with a humidified atmosphere containing 5% CO_2_. The medium was refreshed every 3 days until the cells reached approximately 90% confluence.

### 2.3. Hematoxylin–Eosin (H&E)

A 4% paraformaldehyde solution was used to fix uterine tissues, and the uteri were paraffin-embedded and sectioned (5 μm thickness) for H&E staining. The histological and pathological features were assessed under a microscope (Ni-U, Nikon, Tokyo, Japan).

### 2.4. Protein Extraction and Western Blot Analysis

Uterine tissues and BEECs were lysed with RIPA buffer (KeyGEN BioTECH, Jangsu, China) containing phosphatase and protease inhibitors. Mitochondrial proteins were extracted using a Mitochondrial Protein Extraction Kit (Solarbio, Wuhan, China), and the protein concentration was determined using Bicinchoninic Acid Assay (BCA) Protein Assay Kit (KeyGEN BioTECH, Jiangsu, China). Equal amounts of protein were fractionated by electrophoresis on a 12% or 15% sodium dodecyl sulfate-polyacrylamide gel electrophoresis (SDS-PAGE) and transferred onto polyvinylidene difluoride (PVDF) membranes. Nonspecific binding sites were blocked for 2 h with TBST (50 mmol/L Tris, pH 7.6, 150 mmol/L NaCl, and 0.1% Tween 20) containing 5% BSA. The blots were incubated overnight at 4 °C with the following primary antibodies for detection of: CypD (Proteintech, Wuhan, China, 1:2000), cyto-C (Abcam, Boston, MA, USA, 1:2000), SOD1 (Proteintech, China, 1:2000), p-ERK1/2 (Abways Technology, China, 1:2000), caspase-3 (Abways Technology, Shanghai, China, 1:2000), p-P65 (Abcam, USA, 1:1000), BAX (Proteintech, Wuhan, China, 1:2000), and β-Actin (ZHHC, Shangxi, China, 1:5000). The membranes were then washed with TBST and incubated with horseradish peroxidase-conjugated anti-mouse or anti-rabbit antibody (ZHHC, Xian, China, 1:5000) for 2 h at room temperature. Protein bands were visualized by Image J 1.47V software (National Institutes of Health, Bethesda, MD, USA) and then normalized against β-actin.

### 2.5. ROS Level Determination

BEECs were cultured in 12-well plates (4–8 × 10^5^/well) and treated as indicated. The compound 2,’7’-dichlorofluorescein diacetate (DCFH-DA; Beyotime, Shanghai, China) was used to detect ROS according to the manufacturer’s protocol. BEECs were incubated in serum-free medium containing 10 μmol/L DCFH-DA and then treated with H_2_O_2_ at 37 °C for 5 h; Rosup was used as a positive control. The fluorescence intensity was measured using a fluorescence microscope (Olympus, Tokyo, Japan). The relative intensity of each band was assessed by Image J v 1.47 software.

### 2.6. Transmission Electron Microscopy

BEECs were plated in 12-well plates at 4–8 × 10^5^ cells/well for 24 h and treated as indicated before fixation with 2.5% glutaraldehyde at 4 °C. After washing with PBS, the cells were harvested and fixed with 1% osmic acid at 4 °C for 12 h, washed with distilled water and various concentrations alcohol, followed by two treatments with 100% acetone for 20 min. BEECs were embedded in LR-White, and were cut into 60–80 nm ultrathin sections. The ultrathin sections were dyed with uranium acetate and lead citrate, and examined under an HT7800 transmission electron microscope (Hitachi, Tokyo, Japan).

### 2.7. Mitochondrial Membrane Potential and Apoptosis Detection

The mitochondrial membrane potential (MMP) and apoptosis of BEECs were determined using Mito-Tracker Red CMXRos (Beyotime, Shanghai, China) and Annexin V-FITC Kit (Beyotime, Shanghai, China) according to the manufacturer’s instructions. Briefly, BEECs were plated on 24-well plates (2−5 × 10^5^/well) and incubated with 2 μL Mito-Tracker Red CMXRos, 5 μL Annexin V-FITC, and 5 μL Hoechst 33342 for 30 min. The fluorescence intensity was measured by confocal laser scanning microscopy (Nikon A1Rsi, Tokyo, Japan). The relative intensity was assessed by Image J v 1.47 software.

### 2.8. Detection of Intracellular Adenosine Triphosphate (ATP)

Intracellular ATP levels were measured using an ATP assay kit (Beyotime, Shanghai, China) according to the manufacturer’s instructions. Briefly, the lysates of BEECs were centrifuged at 12,000× *g* for 15 min at 4 °C, and incubated in the ATP-detection buffer. The luminescence signal (RLU) was measured with a luminometer (TUV800, Vienna, Austria).

### 2.9. Cell Viability Assay

Cell viability was measured using the Cell Counting Kit-8 (Cell Counting Kit-8, Beyotime, Shanghai, China) according to the manufacturer’s instructions. The formation of formazan was assessed by determining the optical density (OD) at 450 nm with a microplate spectrophotometer (TUV800, Vienna, Austria).

### 2.10. Detection of Cytoplasmic Ca^2+^

Intracellular calcium concentration was detected by Fluo-4 Calcium Assay Kit (Beyotime, Shanghai, China) according to the manufacturer’s instructions. Briefly, 2–5 × 10^5^/well BEECs were seeded in 24-well plates and then pretreated with or without 10 μM Cyclosporin A (CSA, MedChemExpress, NJ, USA) for 2 h before exposure to H_2_O_2_ and Ca^2+^ detection buffer containing Fluo-4 AM (Beyotime, Shanghai, China) at 37 °C for 30 min. The green (490/525 nm) fluorescence emission was visualized under a laser scanning confocal microscope (Nikon A1Rsi, Nikon, Tokyo, Japan). The relative intensity was assessed by Image J v 1.47 software.

### 2.11. Statistical Analysis

All data analyses were performed using GraphPad Prism 8 (GraphPad InStat Software, San Diego, CA, USA). Differences between two groups were analyzed by Student’s *t*-test. Differences between multiple groups were analyzed by one-way ANOVA. All experiments were repeated at least three times, and all data are presented as means ± standard error of the mean (mean ± SEM). Significance was set at *p* < 0.05.

## 3. Results

### 3.1. Apoptosis-Related Protein Caspase-3, Cyto-C, and mPTP Key Protein CypD Were Significantly Increased in Dairy Cow Uteri with Endometritis

**Figure 1:**
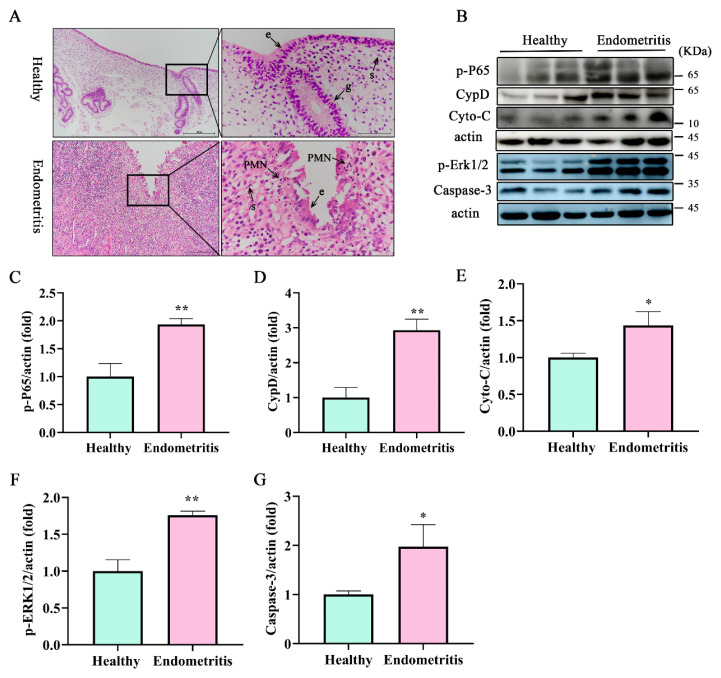
In the endometrium with endometritis, the cells were damaged. (**A**) H&E staining to detect the pathological changes and inflammatory cells infiltration in endometrial layer. Scale bar, 100 μm, 25 μm. (**B**) Inflammatory factor p-P65, mPTP key protein CypD, proapoptotic proteins Cyto, caspase-3, and p-PERK1/2 were detected by Western blot. Relative expression of protein levels of (**C**) p-P65, (**D**) CypD, (**E**) Cyto-C, (**F**) p-ERK1/2, (**G**) caspase-3. e indicates a luminal epithelial cell, s indicates a stroma cell, g indicates a glandular epithelial cell, pmn indicates a polymorphonuclear cell. Data represent mean ± SEM. Statistical significance was set at *p* < 0.05, * *p* < 0.05, ** *p* < 0.01 (unpaired Student’s *t*-test).

The initiation of postpartum inflammatory conditions in the uterus is typically triggered by bacterial contamination of the uterine lumen, leading to an influx of polymorphonuclear cells (PMNs). Uteri of dairy cows were divided into healthy and endometritis groups based on H&E staining and the expression of inflammatory factors. As shown in Figure 1A, epithelial cells in the endometriotic tissues were exfoliated with a large number of inflammatory cells infiltrating the lamina propria of the uterus compared with the healthy group. There was a large amount of PMN infiltration in the endometrium compared with the healthy group. These PMNs are attracted to the uterus through chemokine secretions, and play a pivotal role in the uterine immune response [29,30]. In accordance with our previous study [28], the uteri were categorized into healthy and the endometritis groups. Notably, the inflamed uterine mucus exhibited a significant increase in PMN counts compared to the healthy group (Figure 1A). Furthermore, the endometritis group showed recruitment of lymphocytes into the lamina propria, as well as notable damage to the mucosal layer and basement membrane (Figure 1A). Compared with the healthy group, the expression of the inflammation-related protein p-P65 was higher in the endometritis group (Figure 1B,C, *p* < 0.01). The expression of the mPTP key protein CypD (Figure 1B,D *p* < 0.01) in the endometritis group was significantly increased at the protein level. The expression of apoptosis-related proteins cyto-C (Figure 1B,E *p* < 0.05), caspase-3 (Figure 1B,G *p* < 0.05), *p*-ERK1, and p-ERK2 (Figure 1F,G *p* < 0.01) were significantly increased in the endometritis group compared with the healthy group. These findings indicate that in the endometrium with endometritis the cells were damaged.

### 3.2. Mitochondrial Dysfunction Promotes the Expression of Apoptotic Proteins of BEECs

**Figure 2:**
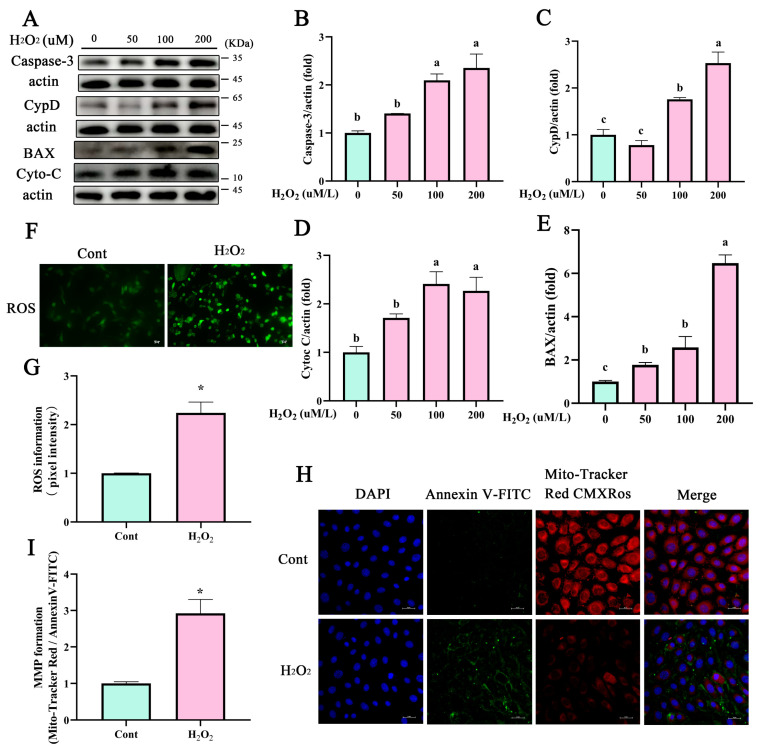
H_2_O_2_ increased apoptosis-associated proteins and mitochondrial dysfunction in BEECs. (**A**–**E**) Western blot analysis of the expression of apoptosis-related proteins caspase-3, BAX, cyto-C, and the mPTP key protein CypD in BEECs treated with various concentrations of H_2_O_2_ (50–200 μM). (**F**,**G**) ROS were detected by fluorescence probes DCFH-DA. (**H**,**I**) The MMP and apoptosis of BEECs were determined using Mito-Tracker Red CMXRos (red) and Annexin V-FITC (green), respectively, in BEECs treated with 200 μM H_2_O_2_. Scale bar, 25 μm. All experiments were performed at least three times. Differences between two groups were analyzed by Student’s *t*-test. Differences between multiple groups were analyzed by one-way ANOVA. Different letters indicate significance between two groups. Data represent mean ± SEM. Significance was set at *p* < 0.05, * *p* < 0.05.

BEECs were isolated from a healthy uterus of a dairy cow according to our previous study [28]. BEECs were exposed to various concentrations of H_2_O_2_ (50, 100, and 200 μM) to induce oxidative stress as a model of bovine endometritis. Compared with the control (0 μmol/L H_2_O_2_) group, expression of the mPTP constituent protein CypD was significantly increased in H_2_O_2_-treated BEECs (Figure 2A,C, *p* < 0.05). Additionally, expression levels of the apoptosis-related proteins caspase-3, cyto-C, and BAX were higher in the H_2_O_2_-treated groups compared with that in the control group (Figure 2A,D,E,I, *p* < 0.05). The fluorescence intensity associated with ROS levels was also greater in BEECs treated with H_2_O_2_ compared with that in the control group (Figure 2F,G, *p* < 0.05). The MMP formation of BEECs was detected after treatment with H_2_O_2_; compared with the control group, the MMP was significantly decreased (Figure 2H,I, *p* < 0.05). 

### 3.3. ROS Removal by NAC Prevented Mitochondrial Vacuolization in BEECs

**Figure 3:**
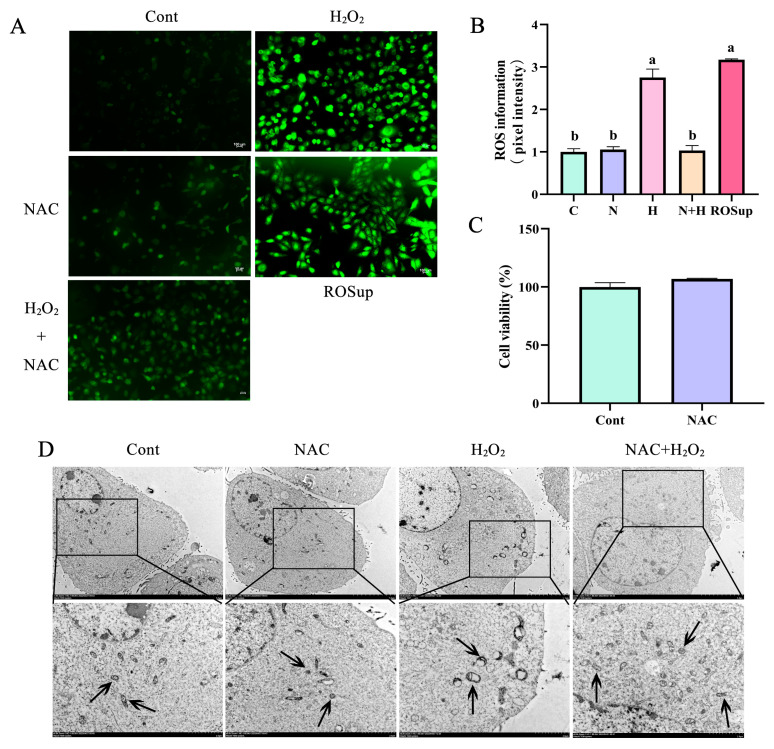
ROS removal by NAC treatment significantly reduced the expression of mitochondria-dependent apoptotic proteins. (**A**,**B**) NAC was used to remove ROS induced by 200 μM H_2_O_2_. Scale bar, 100 μm. (**C**) CCK-8 assay of the cytotoxic effects of NAC (60 μM) on BEECs. (**D**) Mitochondrial morphology in BEECs subjected to H_2_O_2_ and NAC treatments, as well as in untreated cells examined by transmission electron microscopy. Scale bar, 5 μm and 2 μm. The arrow points to the mitochondria. H, H_2_O_2_; N, NAC; ROSup was a positive control reagent that significantly enhance the intracellular ROS. All experiments were performed at least three times. Data represent mean ± SEM. Statistical significance was set at *p* < 0.05. Differences between two groups were analyzed by Student’s *t*-test. Differences between multiple groups were analyzed by one-way ANOVA. Different letters indicate significance between two groups.

To evaluate the effect of ROS on mitochondrial dysfunction, NAC was used to remove ROS. CCK-8 results showed that NAC incubation had no significant effect on BEEC proliferation compared with the control group (Figure 3C). Incubation with H_2_O_2_ significantly increased ROS levels in BEECs, and this effect was significantly (*p* < 0.05) inhibited by NAC treatment (Figure 3A,B). H_2_O_2_ treatment significantly increased ROS levels in BEECs compared with the control group (Figure 3A,B). A large number of mitochondria exhibited vacuolation, swelling, and ridge disappearance after incubation with 200 μM H_2_O_2_ in BEECs. After removal of ROS by NAC treatment, H_2_O_2_-induced mitochondrial damage was alleviated and the number of swelling mitochondria was significantly reduced; mitochondrial damage was significantly reduced compared with the control group (Figure 3A,B). These results indicated that ROS are the main cause of mitochondrial damage in BEECs.

### 3.4. ROS Removal by NAC Restores Mitochondrial Membrane Potential and Energy Synthesis to Normal Levels

**Figure 4:**
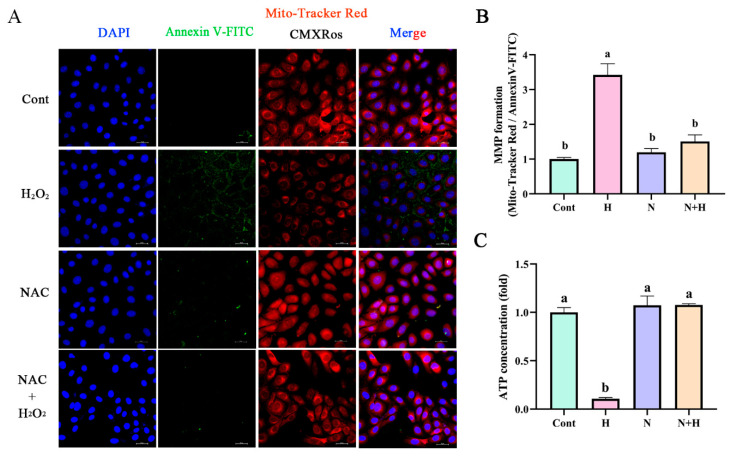
ROS removal by NAC inhibited the effects of H_2_O_2_ treatment on the mitochondrial function. (**A**,**B**) The MMP and apoptosis of BEECs pretreated with or without NAC followed by challenge with 200 μM H_2_O_2_ determined using Mito-Tracker Red CMXRos (red) and Annexin V-FITC (green). Scale bar, 25 μm. (**C**) ATP synthesis was detected in BEECs pretreated with 60 μM NAC in the absence or presence of H_2_O_2_. H, H_2_O_2_; N, NAC. All experiments were performed at least three times. Data represent mean ± SEM. Statistical significance was set at *p* < 0.05. Differences between multiple groups were analyzed by one-way ANOVA. Different letters indicate significance between two groups.

The decreased MMP induced by H_2_O_2_ was significantly enhanced by pretreatment of 60 μM NAC in BEECs. (Figure 4A,B, *p* < 0.05). The effects of H_2_O_2_ treatment on ATP levels in BEECs were inhibited after the removal of ROS by NAC (Figure 4C, *p* < 0.05).

### 3.5. ROS Removal by NAC Treatment Significantly Reduced the Expression of Mitochondria-Dependent Apoptotic Proteins

**Figure 5:**
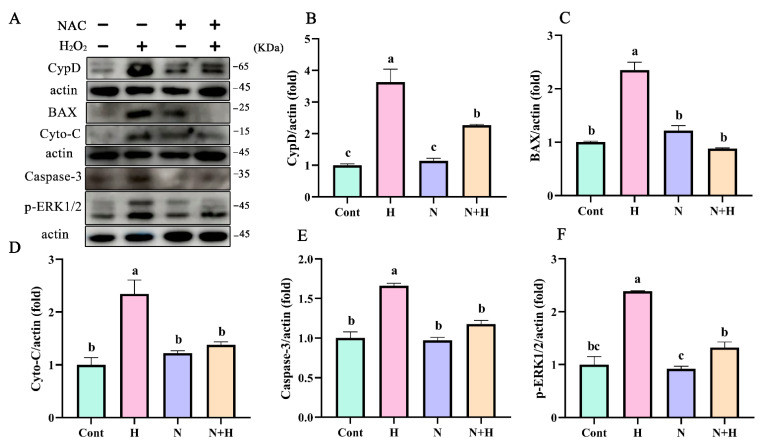
ROS removal by NAC treatment significantly reduced the expression of mitochondria-dependent apoptotic proteins. (**A**,**B**) Western blot analysis the expression of mPTP key protein CypD. (**A**,**C**,**D**,**E**) Western blot analysis of the expression of mitochondria-dependent apoptotic proteins BAX, cyto-C, caspase-3, and p-ERK1/2 expression levels in BEECs pretreated with 60 μM NAC in the absence or presence of H_2_O_2_. All experiments were performed at least three times. Data represent mean ± SEM. Statistical significance was set at *p* < 0.05. Differences between multiple groups were analyzed by one-way ANOVA. Different letters indicate significance between two groups.

To evaluate effect of ROS on mitochondrial dysfunction, NAC was used to remove ROS. Activation of the key mPTP protein CypD by H_2_O_2_ was inhibited after ROS removal by NAC treatment (Figure 5A,B). In addition, expression of the mitochondrial proapoptotic protein BAX was significantly decreased after ROS removal (Figure 5A,C, *p* < 0.05). Western blot was used to analyze of the mitochondrial pathway-related apoptosis protein cyto-C. Compared with the H_2_O_2_ treated group, the expression of cyto-C was significantly decreased after ROS removal by NAC treatment (Figure 5A,D, *p* < 0.05). Additionally, after ROS removal, the expression levels of the apoptosis executioner protein caspase-3 (Figure 5A,E, *p* < 0.05) and *p*-ERK1/2 (Figure 5A,F, *p* < 0.05) were decreased compared with the control group. These results indicated that ROS are the main cause of mitochondrial damage in BEECs.

### 3.6. mPTP Inhibition Prevented the Release of Cyto-C and Ca ^2+^ into the Cytoplasm

**Figure 6:**
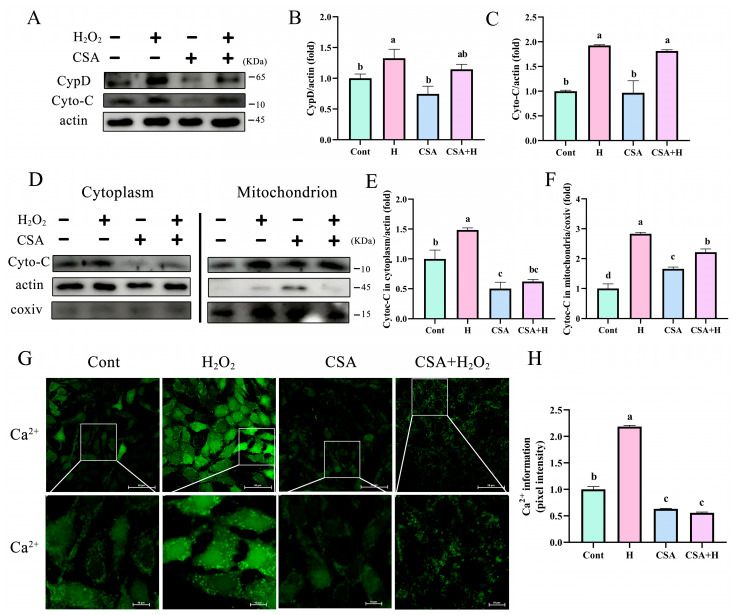
Inhibiting mPTP effectively prevented H_2_O_2_-induced mitochondrial damage and apoptosis in BEECs. BEECs were pretreated with 5 μM CSA in the absence or presence of H_2_O_2_. (**A**–**C**) Western blot analysis of the total protein expression levels of CypD and cyto-C. (**D**–**F**) BEECs were pretreated with 5 μM CSA in the absence or presence of H_2_O_2_. Western blot analysis of mitochondrial and extramitochondrial protein expression levels of CypD and cyto-C. (**G**,**H**) Ca^2+^ levels monitored using Fluo-4 AM. H, H_2_O_2_; N, NAC; CSA, cyclosporin A. Scale bar, 10 μm. All experiments were performed at least three times. Data represent mean ± SEM. Statistical significance was set at *p* < 0.05. Differences between multiple groups were analyzed by one-way ANOVA. Different letters indicate significance between two groups.

CypD is a mitochondrial protein involved in the regulation of the mPTP, which is a key factor in the pathogenesis of mitochondrial dysfunction. Cyclosporin A (CSA), which blocks mPTP opening, significantly (*p* < 0.05) reduced the expression of CypD induced by H_2_O_2_ in BEECs (Figure 6A,B, *p* < 0.05). However, CSA had no significant effects on the expression of cyto-C in BEECs treated with H_2_O_2_ compared with the control group (Figure 6A,C, *p* < 0.05). Furthermore, the levels of cyto-C induced by H_2_O_2_ were significantly decreased in the cytoplasm when mPTP opening was inhibited by CSA (Figure 6D–F, *p* < 0.05). Compared with the levels in the mitochondria of BEECs treated with H_2_O_2_ alone, cyto-C levels were significantly increased after treatment with CSA to inhibit mPTP opening (Figure 6D–F, *p* < 0.05). These findings indicate that cyto-C efflux from the mitochondria to the cytoplasm was significantly decreased after mPTP inhibition. Furthermore, H_2_O_2_ significantly induced mitochondrial calcium efflux and inhibition of mPTP opening by CSA treatment, preventing Ca^2+^ outflow into the cytoplasm (Figure 6G,H, *p* < 0.05). These results indicated that in mitochondria-dependent apoptosis processes, proapoptotic protein cyto-C and Ca^2+^ flow to the cytoplasm through CypD-controlled mPTP to induce BEEC apoptosis.

## 4. Discussion

Oxidative stress and inflammation are two interrelated processes that occur in response to a variety of factors [37], including infection, injury, and chronic diseases. In our previous study, dairy cows with endometritis exhibited oxidative damage, evidenced by elevated levels of ROS in mitochondria, leading to damage in BEECs through mitochondria-dependent pathways [7]. As reported, epithelial cells are particularly susceptible to oxidative damage due to their exposure to various environmental factors such as ultraviolet irradiation, air pollutants, and chemicals [38]. ROS accumulation has been reported to induce oxidative damage and endoplasmic reticulum stress in immortalized human keratinocytes [39]. Oxidative damage to epithelial cells results in a variety of pathological conditions, including inflammation, tissue injury, cancer, and Alzheimer’s disease [40,41]. Removing ROS by antioxidants is shown to enhance anti-inflammatory, antidiabetic, and anticancer properties, improving the gut microbiome [42]. Cyto-C is a protein encoded by the nucleus gene, and plays a vital role in the mitochondrial electron transport chain [13]. Under normal circumstances, it exists in the space between the inner and outer membrane of mitochondria [43]. In pathologic conditions, such as ischemia-reperfusion, phosphorylations are lost, leading to maximum electron transport chain flux, MMP hyperpolarization, excessive ROS generation, and the release of cyto-C [43]. The release of cyto-C from mitochondria plays an important role in apoptosis. The stimulation of an apoptosis signal causes cyto-C to be released from the mitochondria into the cytoplasm, mediating apoptosis [44].

In the present study, high levels of ROS in BEECs induced high expression of pro-apoptosis-related proteins cytochrome C and caspase 3. Mitochondria are the main place to produce ROS. During oxidative stress, the normal function of mitochondria will be seriously damaged [45]. Under conditions of chronic oxidative stress, the defense mechanisms of epithelial cells become overwhelmed, leading to cellular damage and dysfunction [46]. H_2_O_2_ was used to establish a model of endometritis in vitro. We found that the expression of mitochondria-dependent proapoptotic protein was significantly increased. with the increase of H_2_O_2_. We also found that mitochondrial dysfunction such as ATP synthesis was blocked, and mitochondrial membrane potential decreased. In accordance with these reports, we found a higher degree of inflammatory cell infiltration in the uteri with oxidative stress. These findings are consistent with a previous report describing increased oxidative damage in uteri with endometritis [47]. Based on our results, we found that mitochondrial dysfunction was accompanied with an inflammatory response in BEECs.

CypD, which is a mitochondrial protein involved in the regulation of mPTP, is a key factor in the pathogenesis of mitochondrial dysfunction [48] and has been implicated in the development of inflammation [29]. Mounting evidence implicates a decline in mitochondrial function due to increased opening of mPTP [49]. CypD has been shown to be involved in opening of mPTP, leading to mitochondrial dysfunction and cell death. Studies have shown that inhibition of CypD reduces the severity of inflammation in MC3T3-E1 cells by reducing apoptosis and oxidative stress [29]. Cyto-C is a protein that is normally found in the mitochondrial intermembrane space. During apoptosis, cyto-C is released into the cytosol where it binds to the protein Apaf-1, forming a complex called the apoptosome [50]. In accordance with our findings, inhibition of CypD by CSA significantly reduced the levels of the proapoptotic protein cyto-C in the cytoplasm of BEECs. The apoptosome then activates caspase-9, which in turn activates caspase-3, which initiates the apoptotic process [44]. In the current study, we found that the levels of CypD and cyto-C proteins were significantly increased in uteri with endometritis and in BEECs treated with H_2_O_2_ compared with those in the healthy group.

**Figure 7:**
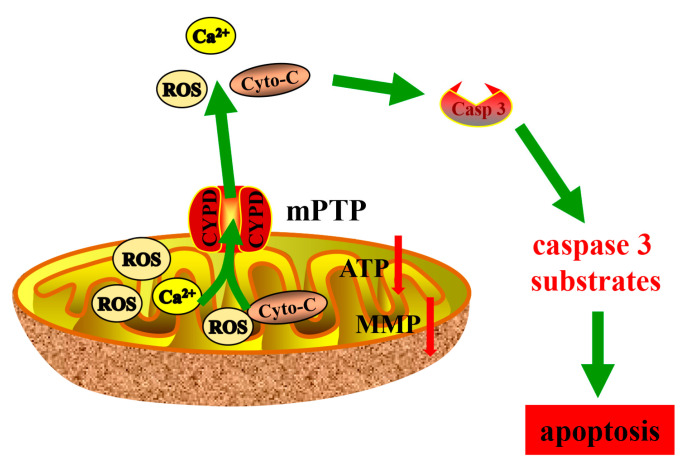
A schematic model of mitochondrial dysfunction-induced BEEC apoptosis.

In the early stages of mitochondria-dependent apoptosis, the MMP decreases, while the mitochondrial outer membrane permeability increases, along with high expression levels of cyto-C [51]. These two major changes facilitate the release of soluble membrane proteins from the mitochondria. In a normal state, the mPTP is closed, preventing the release of cyto-c and Ca^2+^ into the cytoplasm [52]. The inactive mPTP (closed) prevents the uncontrolled influx of protons and solutes, which is important for maintaining the electrochemical gradient and oxidative phosphorylation [53]. Evidence suggests that brief mPTP opening plays an important role in maintaining mitochondrial homeostasis [54]. The imbalance of Ca^2+^ or mitochondrial signaling leads to functional abnormalities, cell damage, and even cell death, leading to muscle dysfunction or heart disease [55]. We found that H_2_O_2_-induced disturbances such as MMP decreased, and ROS increased in BEECs (Figure 7). However, the cytoplasmic Ca^2+^ concentration was significantly reduced after CSA-mediated inhibition of CypD, suggesting that the mPTP is essential for Ca^2+^ regulation, and an imbalance in the cellular levels of Ca^2+^ is a key factor that induces mitochondrial dysfunction and cyto-C release. CypD is located in the mitochondrial matrix and is a key promoter of mPTP opening, regulating the permeability of mPTP in response to various stress stimuli [56,57]. CypD overexpression disrupts its recruitment to mPTP channels, leading to continuous pore opening and widespread swelling of mitochondria [58]. In this study, we demonstrated that CSA-mediated inhibition of CypD significantly diminished the flow of cyto-C and Ca^2+^ from mitochondria to the cytoplasm (Figure 7). Recent studies have shown that CypD promotes ROS production by regulating mPTP opening [29], leading to a decrease in MMP and an increase in electron leakage in the electron transfer chain (ETC). CypD has been shown to limit mitochondrial ROS production and protect against ischemia-reperfusion injury in the heart and brain [59,60]. Mitochondria are the main targets of excessive ROS, which induce the opening of the mPTP, leading to the release of Ca^2+^, cyto-C, and apoptosis-inducing factors, which activate caspase-9 and caspase-3/6/7 through activating the cysteine aspartate-specific protease [61]. Moreover, ROS uncouples the mitochondrial ETC, downregulates ATP production levels, upregulates the expression of the proapoptotic protein Bax, and ultimately causes rupture of the mitochondrial outer membrane, resulting in cell apoptosis [62]. In our study, ATP synthesis was significantly reduced and Bax expression was significantly increased under oxidative stress. In BEECs pretreated with NAC to scavenge ROS, we found that ATP synthesis returned to normal levels, and there was no significant change in the expression of proapoptotic proteins compared with those in the control group. We found that the key upstream signaling pathways of mitochondrial dysfunction in BEECs under oxidative stress mainly involved disturbances in Ca^2+^ homeostasis, leading to Ca^2+^ overload and subsequent activation of the mPTP due to altered low conductance permeability (Figure 7).

## 5. Conclusions

The expression of mitochondrial proapoptotic protein was significantly increased in uteri with endometritis. In the context of the inflammatory response mediated by oxidative stress, mitochondria exhibited mitochondrial dysfunction in BEECs in vitro. Mitochondrial dysfunction of BEECs includes diminished MMP, disrupted calcium homeostasis, diminished ATP synthesis, excessive expression, and continued activation of mPTP. Cyto-C released from the injured mitochondria into the cytoplasm through mPTP activated apoptosis executive protein caspase3. Ultimately, this intricate series of events results in mitochondrial damage-dependent apoptosis in BEECs. Thus, our study is the first to demonstrate that apoptosis is induced in BEECs via a ROS-CypD-cyto-C-dependent mechanism, which represents a potential therapeutic target for oxidative injury-related endometritis.

## Data Availability

Data are contained within the article.
